# Awareness, Knowledge, and Attitudes Regarding Basic Life Support Among the Population With Relatives Suffering From Heart Diseases in the Al-Qassim Region, Saudi Arabia

**DOI:** 10.7759/cureus.31530

**Published:** 2022-11-15

**Authors:** Sami M Alrasheedi, Mousa N Alrashdi, Khalid F Almutairi, Abdulmgeed F Alruways, Ibrahim N Almutairi, Sultan N Alfehaid, Ohud A Alrashdi, Ahmad Alkhdairi, Ahmed S Alrashidi, Yasser N Aloraini

**Affiliations:** 1 Department of Medicine, Unaizah College of Medicine and Medical Sciences, Qassim University, Unaizah, SAU; 2 Internal Medicine, Unaizah College of Medicine and Medical Sciences, Qassim University, Unaizah, SAU; 3 Department of Medicine, College of Medicine, Shaqra University, Dawadmi, SAU; 4 Internal Medicine, College of Medicine, King Saud University, Riyadh, SAU; 5 Primary Care Center, Qassim Health Cluster, Qassim, SAU; 6 Otolaryngology, King Salman Bin Abdulaziz Medical City, Madinah Health Cluster, Madinah, SAU; 7 Department of Home Health Care, Bukairyyah General Hospital, Qassim Health Cluster, Bukairyyah, SAU

**Keywords:** acls, cpr, heart disease, bls, basic life support

## Abstract

Background and objective

Basic Life Support (BLS) is critical because it keeps patients with life-threatening illnesses or injuries alive and maintains viability until a team of paramedics or hospital staff can provide expert care. There are many events that can result in serious injury and cause a person to stop breathing. BLS awareness among the population who have relatives with heart diseases greatly increases their confidence to act quickly when necessary and reduces their level of hesitation. In this study, we aimed to evaluate the level of clinical competence in the population who have relatives with heart diseases for them to recognize and respond to individuals in need of BLS in the Qassim region of Saudi Arabia.

Methodology

We conducted a quantitative, observational, and analytical cross-sectional study to achieve our objective. The targeted population involved only Saudis. The study was conducted electronically using social network apps in the Al-Qassim region of Saudi Arabia. The questionnaire evaluated if the subject had a family member with heart disease. Data collected included sociodemographic characteristics and knowledge and awareness about BLS as related to specific objectives.

Results

Of the 414 participants, 58.8% were females; 33.3% were between the ages of 40-49 years, and 30.7% were between 18-29 years old. The prevalence of participants who reported participating in cardiopulmonary resuscitation (CPR) training was 19.8%. The main reasons for not participating in these training were a lack of knowledge about the courses (60.5%), being too busy (16.2%), and thinking that they did not need them (12.3%). Being younger than 29 years in age was one of the factors associated with participating in CPR training [odds ratio (OR): 11.85, 95% confidence interval (CI): 1.54-91.42, p=0.017] versus those aged over 59 years. Gender was significantly associated with the rate of participation in CPR training: females had significantly lower rates of participation than males (15.2% vs. 25.7%, OR: 0.52, 95% CI: 0.32-0.84, p=0.008). Of note, 25.5% of the participants had adequate knowledge regarding CPR. Having trained in CPR was significantly associated with a higher level of knowledge among the participants (1.82-fold) (OR: 1.82, 95% CI: 1.08-3.06, p=0.023).

Conclusion

Based on our findings, there is limited awareness and training related to CPR among people with relatives suffering from cardiac diseases in the Qassim region of Saudi Arabia. This may be associated with higher rates of morbidity and mortality related to heart diseases in the region.

## Introduction

Public awareness is of great importance regarding life-saving information and the skills associated with it. Many countries emphasize imparting such skills in educational institutions and workplaces. The incidence of deaths due to sudden cardiac arrest remains very high worldwide [1,2]. Basic Life Support (BLS) helps 50,000 people per year to survive until professional help arrives, especially when BLS measures are performed properly. Out-of-hospital cardiac arrest (OHCA) is associated with more than 350,000 deaths per year in Europe alone [3]. In the US, the mortality rate related to OHCA is more than 90% and accounts for 276,000 deaths annually [4].

The performance of BLS is usually conducted in chains, and it can be improved through educational programs. Patient outcomes improve when BLS is performed by a layman before professional help arrives [5]. However, this requires high motivation and awareness among the public. It can be achieved by increasing public knowledge, attitudes, and understanding regarding the application of BLS techniques. There is a sequence of steps that constitute the chain of survival including early approaches to cohesive medical emergencies, early initiation of BLS, early defibrillation, and advanced life support (ALS) [6].

Life-threatening emergencies can happen anytime and anywhere. The lack of training and inability to deal with these emergencies can lead to both medical and legal consequences. BLS skills including cardiopulmonary resuscitation (CPR) in a quick and efficient manner improve survival after cardiac arrest. Sudden cardiac arrest is the most common cause of death worldwide. Survival rates vary greatly between various societies [7]. Initiating CPR has reduced morbidity and mortality among those who experience sudden cardiac arrest [7-9]. Previous studies in Arizona have shown that statewide public awareness campaigns for CPR led to increased rates of CPR being performed. The rates of bystander-performed CPR went up from 28.2% to 39.9% and improved OHCA survival rates from 3.7% to 9.8% [10]. The number of OHCA cases in the United States is approximately 300,000, with an associated mortality rate of 92% [11]. The chances of survival doubled if BLS was performed by the first person to intervene and with the use of an automated external defibrillator [11].

The difficulty in performing CPR among bystanders in developed countries is due to insufficient knowledge or training, lack of skills, lack of confidence, and fear of prosecution for providing improper care [12]. Communities need to acquire adequate knowledge and understanding of BLS through in-person or online training [13]. There are several ways/measures to increase the success of CPR and BLS in any emergency; these measures can increase the general knowledge and understanding regarding the practical application of BLS interventions for successful post-emergency outcomes. Knowledge and community perceptions of BLS in Saudi Arabia have not yet been assessed. In light of this, the aim of this study is to evaluate the level of clinical competence of the population with relatives suffering from heart diseases to recognize and respond to individuals who need BLS in the Qassim region of Saudi Arabia.

## Materials and methods

Study design and setting

This was a quantitative, observational, and analytical cross-sectional study. The targeted population involved only Saudis. The study was conducted electronically using social network apps in the Al-Qassim region of Saudi Arabia. The questionnaire determined if the subject had a family member with heart disease.

Sample size

The sample size was calculated via the following formula: n=Z2*pq*DE/d2; where n: sample size; Z: standard deviation=1.96; p: prevalence=16; q: 1-p; DE: design effect=2; and d: error accepted=0.05.

The sample size was calculated to be 380. The participants were selected by non-probability convenience sampling technique.

Inclusion and exclusion criteria

We included Saudi nationals aged ≥18 years, who were mentally competent, had patients with heart diseases as relatives, and lived in the Al-Qassim region. We excluded those who were <18 years of age and were relatives of patients with non-heart diseases.

Data collection methods

A semi-structured questionnaire was used for collecting data. This data collection tool was developed by Dr. Jingjing Huang and his team [14]. Data collected included sociodemographic characteristics and knowledge and awareness about BLS as related to specific objectives.

Data analysis plan

SPSS Statistics version 26.0 (IBM Corp., Armonk, NY) was used for statistical analysis. The frequency and percentiles were used for descriptions of categorical variables. Mean and standard deviations were used to describe ongoing variables. The Chi-squared test, t-test, and analysis of variance (ANOVA) were used to evaluate the difference and relationship between variables. The threshold of significance (p-value) was set at 0.05 with a 95% confidence interval (CI).

Ethical considerations

The study was conducted after obtaining ethical approval from the Qassim University Ethics Committee. All participants voluntarily agreed to participate in the study. We ensured that no participant would experience stress, discomfort, worry, or a loss of self-esteem; there was no invasion of their privacy. All information obtained from the participants remained confidential. All participants gave informed written consent.

## Results

We initially enrolled 851 participants, but 437 of them were excluded because they did not have a relative with cardiac conditions or were not from the Qassim region. Thus, the final sample consisted of 414 participants; 58.8% of them were females, 33.3% were aged between 40-49 years, and 30.7% were between 18-29 years old. Of note, 76.6% of them had a college degree or more while 26.3% worked in the educational sector. Another 25.4% were government employees and 18.6% were students. Two-thirds of the sample reported living in the city at the time of the study, and 46.4% had monthly incomes of 10,000-20,000 Saudi Riyal (SR) (Table 1).

**Table 1:** General demographic characteristics of the study participants

Characteristics			
		N	%
Gender	Male	183	44.2%
	Female	231	55.8%
Age (years)	18–29	127	30.7%
	30–39	60	14.5%
	40–49	138	33.3%
	50–59	70	16.9%
	>59	19	4.6%
Educational level	Primary or lower	2	0.5%
	Middle school	16	3.9%
	High school	79	19.1%
	College or higher	317	76.6%
Profession	Healthcare	28	6.8%
	Education	109	26.3%
	Social services	6	1.4%
	Government employee	105	25.4%
	Farmer	5	1.2%
	Commercial	15	3.6%
	Student	77	18.6%
	Others	69	16.7%
Current residence	City	280	67.6%
	Governorate town/village	134	32.4%
Total household income (Saudi Riyal)	<10,000	117	28.3%
	10,000–20,000	192	46.4%
	>20,000	105	25.4%

Among the participants, 33.3% reported that the relative with heart conditions was their father, followed by uncles or aunts (22.0%) or children (15.5%). Hypertension was the most common heart condition known in those relatives (23.7%), followed by arterial obstruction or narrowing (18.9%) and aortic dissection (14.0%); 12.6% did not know the type of heart condition their relatives had. Another 47.9% of the participants reported that their relatives had visited the hospital for heart disease two to three times in the past year while 30.8% reported only one visit in the last year. As for surgeries for heart disease, 41.5% reported that their relatives had undergone surgery only once, while 18.6% reported two to three surgeries and 8.2% reported that their relatives never had surgery (Table 2).

**Table 2:** Medical characteristics of patients with heart conditions

Characteristics		N	%
Relationship between you and the patient with heart disease	Father	138	33.30%
	Uncle/aunt	91	22.00%
	Sibling	58	14.00%
	Children	64	15.50%
	Wife	22	5.30%
	Friend	41	9.90%
What kind of heart disease does the relative have?	Congenital heart disease	31	7.50%
	Valvular heart disease	54	13.10%
	Aortic dissection	58	14.00%
	Arrhythmia	24	5.80%
	Hypertension	98	23.70%
	Arterial obstruction or narrowing	78	18.90%
	Unclear	52	12.60%
	Others	18	4.40%
How many times has your relative visited the hospital in the last year (for heart disease)?	1	123	30.80%
	2–3	191	47.90%
	>3	85	21.30%
How many heart surgeries has your family member undergone (for heart disease)?	0	34	8.20%
	1	172	41.50%
	2–3	77	18.60%
	>3	9	2.20%
	Unclear	122	29.50%

**Figure 1:**
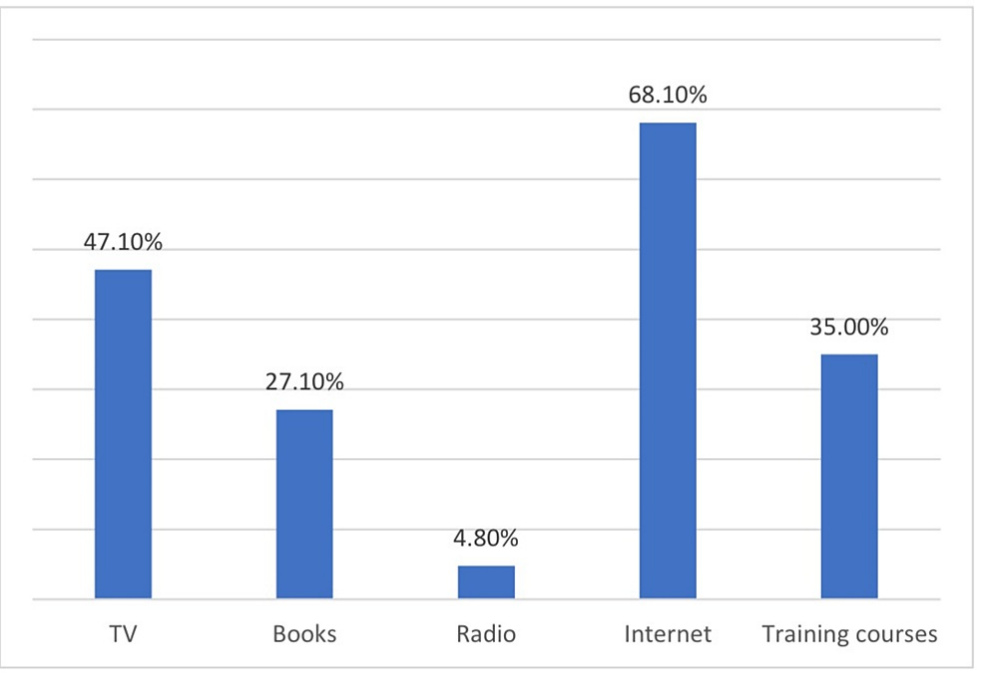
The source of information regarding CPR CPR: cardiopulmonary resuscitation

The internet was the most common source of information on CPR for more than two-thirds of the participants (68.1%), followed by TV (47.1%), training courses (35.0%), and books (27.1%) (Figure 1).

The proportion of participants who reported participating in CPR training was 19.8%. The main reasons for not participating in these training were a lack of knowledge about the courses (60.5%), being too busy (16.2%), and thinking that they did not need them (12.3%). Moreover, 94.9% of the participants reported that it is necessary to be trained and learn about CPR, and 86.5% stated they would like to attend these courses if they were provided free of cost. Moreover, 37.7% thought that CPR training courses should be conducted in companies, 34.8% stated that these should be conducted in schools, 17.1% endorsed conducting them in hospitals, and 10.4% reported that the skills should be acquired by self-study (Table 3).

**Table 3:** Participation in CPR training CPR: cardiopulmonary resuscitation

CPR training		N	%
Have you participated in CPR training?	No	332	80.2%
	Yes	82	19.8%
Why did you not get trained in CPR?	Too busy	54	16.2%
	Not interested	37	11.1%
	Do not think I need it	41	12.3%
	Have not heard of training courses	202	60.5%
It is necessary to be trained in and learn about CPR	No	21	5.1%
	Yes	393	94.9%
If there are free CPR training courses, would you like to attend them?	No	56	13.5%
	Yes	358	86.5%
Where do you think CPR training courses should be conducted?	Hospital	71	17.1%
	Company	156	37.7%
	School	144	34.8%
	Self-study	43	10.4%

Being younger than 29 years in age was one of the factors associated with higher participation in CPR training [odds ratio (OR): 11.85, 95% CI: 1.54-91.42, p=0.017] versus those who were older than 59 years. In general, younger age was significantly associated with a higher CPR training participation rate (p<0.001). Moreover, gender was significantly associated with the rate of participation: CPR training among females was significantly lower than among males (15.2% vs. 25.7%, OR: 0.52, 95% CI: 0.32-0.84, p=0.008). No significant difference was found between participants regarding participation rates in terms of their current residence (p=0.357), total monthly income (p=0.135), relationship with the patient (0.437), or the number of surgeries the patient had undergone (p=0.066) (Table 4).

**Table 4:** Predictors of participation in CPR training *Statistically significant CPR: cardiopulmonary resuscitation

Variables		Have you participated in CPR training?					
		No		Yes			
		N	%	N	%	OR (95% CI)	P-value
Gender	Male	136	74.3%	47	25.7%	Reference	
	Female	196	84.8%	35	15.2%	0.52 (0.32–0.84)	0.008*
Age (years)	18–29	82	64.6%	45	35.4%	11.85 (1.54–91.42)	0.017*
	30–39	44	73.3%	16	26.7%	6.55 (0.81–53.10)	0.078
	40–49	124	89.9%	14	10.1%	2.03 (0.25–16.4)	0.516
	50–59	64	91.4%	6	8.6%	1.69 (0.19–14.94)	0.651
	>59	18	94.7%	1	5.3%	Reference	
Current residence	City	221	78.9%	59	21.1%	1.29 (0.76–2.20)	0.357
	Governorate town/village	111	82.8%	23	17.2%	Reference	
Total household income (Saudi Riyal)	<10,000	95	81.2%	22	18.8%	Reference	
	10,000–20,000	162	84.4%	30	15.6%	0.80 (0.44–1.47)	0.135
	>20,000	75	71.4%	30	28.6%	1.73 (0.92–3.34)	0.087
Relationship between you and the patient with heart disease	Father	107	77.5%	31	22.5%	1.03 (0.44–2.39)	0.949
	Uncle/aunt	65	71.4%	26	28.6%	1.42 (0.6–3.39)	0.437
	Sibling	51	87.9%	7	12.1%	0.49 (0.17–1.44)	0.125
	Children	60	93.8%	4	6.3%	0.24 (0.07–0.83)	0.610
	Wife	17	77.3%	5	22.7%	1.05 (0.30–3.62)	0.948
	Friend	32	78.0%	9	22.0%	Reference	
How many times has your family member visited the hospital in the last year (for heart disease)?	1	90	73.2%	33	26.8%	2.23 (1.08–4.63)	0.030*
	2–3	157	82.2%	34	17.8%	1.32 (0.64–2.69)	0.458
	>3	73	85.9%	12	14.1%	Reference	
How many heart surgeries has your family member undergone (for heart disease)?	0	22	64.7%	12	35.3%	1.92 (0.84–4.37)	0.120
	1	144	83.7%	28	16.3%	0.68 (0.38–1.23)	0.127
	2–3	65	84.4%	12	15.6%	0.65 (0.31–1.37)	0.132
	>3	6	66.7%	3	33.3%	1.76 (0.41–7.50)	0.453
	Unclear	95	77.9%	27	22.1%	Reference	

Regarding knowledge about CPR, 25.5% of the participants had adequate knowledge about CPR (able to answer three or more out of four questions correctly). No difference was found between the two genders in terms of knowledge about CPR; however, females seemed to have a slightly higher level of knowledge (OR: 1.23, 95% CI: 0.79-1.93, p=0.370). Younger participants were associated with slightly better knowledge, but this was not significant between any of the age groups. No significant difference was found between participants regarding participation rates in terms of their current residence, total monthly income, relationship with patients, or the number of surgeries the relative had undergone. Having attended a CPR course was significantly associated with higher knowledge among the participants (1.82-fold) (OR: 1.82, 95% CI: 1.08-3.06, p=0.023); 35.4% of those reported participating in CPR training reported having adequate knowledge versus 23.1% of those with no previous training (Table 5).

**Table 5:** Predictors of good knowledge about CPR *Statistically significant CPR: cardiopulmonary resuscitation

Variables		Knowledge					
		Inadequate		Adequate			
		N	%	N	%	OR (95% CI)	P-value
Gender	Male	138	76.7%	42	23.3%	Reference	
	Female	168	72.7%	63	27.3%	1.23 (0.79–1.93)	0.370
Age (years)	18–29	82	64.6%	45	35.4%	2.06 (0.64–6.57)	0.225
	30–39	44	73.3%	16	26.7%	1.36 (0.39–4.72)	0.637
	40–49	112	83.0%	23	17.0%	0.77 (0.23–2.53)	0.126
	50–59	53	75.7%	17	24.3%	1.20 (0.35–4.12)	0.781
	>59	15	78.9%	4	21.1%	Reference	
Educational level	Primary or lower	1	50.0%	1	50.0%	3.42 (0.21–55.41)	0.394
	Middle school	8	50.0%	8	50.0%	3.42 (1.24–9.44)	0.017*
	High school	54	68.4%	25	31.6%	1.58 (0.92–2.73)	0.096
	College or higher	243	77.4%	71	22.6%	Reference	
Current residence	City	210	75.8%	67	24.2%	0.81 (0.51–1.28)	0.138
	Governorate town/village	96	71.6%	38	28.4%	Reference	
Total household income (Saudi Riyal)	<10,000	79	67.5%	38	32.5%	Reference	
	10,000–20,000	144	76.2%	45	23.8%	0.65 (0.39–1.08)	0.105
	>20,000	83	79.0%	22	21.0%	0.55 (0.30–1.01)	0.855
Relationship between you and the patient with heart disease	Father	104	75.4%	34	24.6%	0.46 (0.22–0.96)	0.742
	Uncle/aunt	65	71.4%	26	28.6%	0.56 (0.26–1.22)	0.117
	Sibling	41	70.7%	17	29.3%	0.59 (0.25–1.36)	0.127
	Children	57	93.4%	4	6.6%	0.1 (0.03–0.33)	0.036*
	Wife	15	68.2%	7	31.8%	0.66 (0.22–1.96)	0.135
	Friend	24	58.5%	17	41.5%	Reference	
How many times has your family member visited the hospital in the last year (for heart disease)?	1	84	68.3%	39	31.7%	1.51 (0.8–2.83)	0.201
	2–3	146	77.7%	42	22.3%	0.93 (0.50–1.72)	0.142
	>3	65	76.5%	20	23.5%	Reference	
How many heart surgeries has your family member undergone (for heart disease)?	0	24	70.6%	10	29.4%	0.92 (0.40–2.12)	0.131
	1	133	78.7%	36	21.3%	0.6 (0.34–1.02)	0.875
	2–3	60	77.9%	17	22.1%	0.63 (0.32–1.21)	0.214
	>3	5	55.6%	4	44.4%	1.77 (0.45–6.96)	0.424
	Unclear	84	68.9%	38	31.1%	Reference	
Have you participated in CPR training?	No	253	76.9%	76	23.1%	Reference	
	Yes	53	64.6%	29	35.4%	1.82 (1.08–3.06)	0.023*

## Discussion

CPR is an important part of the armamentarium of first aid responders. Low levels of awareness and training with respect to CPR were reported among our study participants who had relatives with cardiac diseases. Only 19.8% of the individuals in this study reported receiving CPR-related training. This is similar to the findings of other previous studies in different regions worldwide including China where studies have reported that only 3-25% of the individuals reported having CPR training [14-17]. Moreover, in another study from Hong Kong, the rate of participation in CPR training was 21% [18]. However, the prevalence reported here was significantly lower than reported in developed countries, including two studies conducted in the US that reported a participation rate of 78% and 83% [19,20]. In Poland, the rate was 75% [21]; it was 56.0% and 64.1% in Australia [22,23], 40.3% in Turkey [1], 58% in Japan [24], 28% in Ireland [25], 27% in New Zealand [26], and 29% Jordan [27]. In general, this study showed that Saudi Arabia, particularly the Qassim region, has a lower percentage of trained individuals in CPR compared to other countries. This indicates that more efforts are needed to train the public in life-saving skills, particularly because the participants in our study had relatives with cardiac disorders that increased their exposure to these events. Moreover, the difference in the rate of CPR training in this study versus other studies may be attributed to differences in the promotion of the practice among the general public. In our study, 60.5% of the participants had never even heard of CPR training courses versus 33% in a Chinese study [14] and 9.9% in an Australian study [23].

The main sources of information on CPR among our participants were the internet, TV, and training courses. This is similar to the findings of Teng et al. who reported that most of the participants were introduced to CPR by TV or the internet [14]. Another Saudi study showed that the most common sources of information on CPR were TV and movies [28]. This contrasts with a Jordanian study that showed that the main source of information was schools, universities, and the media [27]. Schools and universities are very important sources of information on life-saving skills. The media can reach a broader population and can cover more people to raise their knowledge; however, this can also lead to misinformation. The inadequate promotion of CPR in Saudi Arabia may cause the information to be invisible to the public. Moreover, Saudi Arabia does not have mandatory first-aid training for non-medical individuals. Some previous studies conducted in the US have shown significant positive outcomes after the implementation of mandatory CPR training. People in states with mandatory training programs were 34% more likely to be currently training than those in states without such a program [29].

We found that younger people and the male gender were associated with a greater chance of receiving CPR training. This is similar to the results of previous studies showing higher income, younger age, and higher educational levels to be associated with a greater chance of receiving CPR training [17,23,30]. We found no significant difference between those living in rural and city regions and between those with different incomes in terms of receiving CPR training. Teng et al. found that living in rural areas with low levels of education and low income was associated with low CPR training rates among relatives of patients with heart diseases [14]. Low participation rates among women and older participants may be attributed to the fact that CPR can be physically demanding, and the elderly and women may have difficulties performing these tasks and hence feel reluctant to learn.

Most of our participants reported not receiving CPR training, but a significant majority showed interest in learning CPR: 86% considered learning CPR as necessary, and 94.9% were willing to take training lessons provided they were free of cost. This indicates that residents of Saudi Arabia showed a high desire for CPR training, which is similar to or even higher than reported in other studies in different countries, including 77% and 92% in China [14,15]. Moreover, a previous study conducted in Saudi Arabia showed that 90% of 947 university students wanted to receive CPR training [31].

Three-quarters of our participants had an inadequate level of knowledge regarding CPR. This low awareness was also reported in some previous studies [32-34]. Moreover, having previous CPR training was a factor affecting the participants’ knowledge. Other factors included age, profession, and education level [14].

Various previous studies have shown that incorporating BLS support measures by trained regular individuals/laypersons has a positive effect on reducing both the rates of mortality and morbidity [35-37]. People receiving CPR from trained individuals have a four-fold greater chance of survival and live a month longer than those who did not receive CPR [38]. Establishing more BLS training centers is essential to improve the survival rates among patients with heart diseases. Even schools and companies could be selected as sites for providing courses and training.

## Conclusions

Based on our findings, there is limited awareness and training regarding CPR among people with relatives suffering from cardiac diseases in the Qassim region of Saudi Arabia. This may lead to higher rates of morbidity and mortality. Raising awareness and training individuals would increase the rates of survival among these patients, and this could be achieved by instituting mandatory training in BLS for employees and students.
